# *N*-[11-(4-Chloro­phen­yl)-11,12-dihydro­benzo[*c*]phenanthridin-6-yl]benzamide

**DOI:** 10.1107/S1600536810043485

**Published:** 2010-10-31

**Authors:** Min Zhang, Xiang-Yang Wu, Liu-Qing Yang

**Affiliations:** a School of Chemistry and Chemical Engineering, Jiangsu University, Zhenjiang, Jiangsu Province 212013, People’s Republic of China; b School of the Environment, Jiangsu University, Zhenjiang, Jiangsu Province 212013, People’s Republic of China; c School of Pharmacy, Jiangsu University, Zhenjiang, Jiangsu Province 212013, People’s Republic of China

## Abstract

There are two independent mol­ecules in the asymmetric unit of the title compound, C_30_H_21_ClN_2_O, which differ slightly in the orientation of the unsubstituted phenyl ring. Inter­molecular C—H⋯π inter­actions stabilize the crystal structure. The crystal studied was found to be a racemic twin. The dihedral angles between the substituted phenyl ring and the benzo[*c*]phenanthridine system are 87.13 (5) and 79.25 (5)° in the two molecules.

## Related literature

There are many useful pharmacological properties of benzo[*c*]phenanthridine derivatives (Clement *et al.*, 2005 ▶). For their anti­tumour activity, see: Stermitz *et al.* (1973 ▶, 1975 ▶); Fang *et al.* (1993 ▶); Suzuki *et al.* (1992 ▶); Kanzawa *et al.* (1997 ▶); Guo *et al.* (2007 ▶); for their anti­microbial activity, see: Nissanka *et al.* (2001 ▶); for their anti inflammatory activity, see: Lenfeld *et al.* (1981 ▶); for their anti­tuberculosis activity, see: Ishikawa (2001 ▶). For the synthesis of the starting material, see: Zhang *et al.* (2008 ▶).
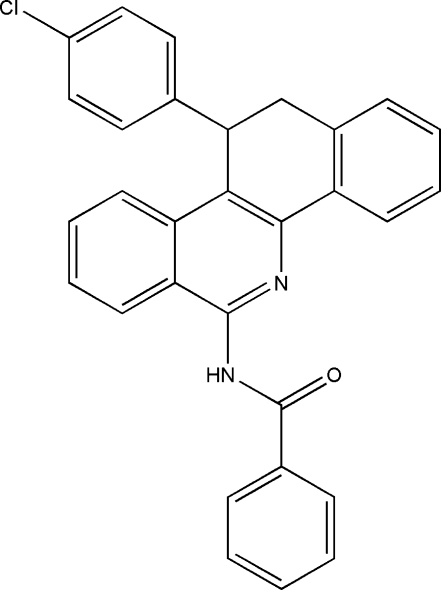


## Experimental

### 

#### Crystal data

C_30_H_21_ClN_2_O
*M* *_r_* = 460.94
Orthorhombic, 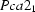

*a* = 26.567 (5) Å
*b* = 9.6752 (19) Å
*c* = 17.329 (4) Å
*V* = 4454.5 (15) Å^3^
*Z* = 8
Mo *K*α radiation
μ = 0.20 mm^−1^
*T* = 113 K
0.20 × 0.18 × 0.10 mm

#### Data collection

Rigaku Saturn724 CCD diffractometer
Absorption correction: multi-scan (*CrystalClear*; Rigaku, 2009 ▶) *T*_min_ = 0.961, *T*_max_ = 0.980
27069 measured reflections
8288 independent reflections
7795 reflections with *I* > 2σ(*I*)
*R*_int_ = 0.038

#### Refinement

*R*[*F* ^2^ > 2σ(*F* ^2^)] = 0.040
*wR*(*F* ^2^) = 0.103
*S* = 1.06
8288 reflections
613 parameters
1 restraint
H-atom parameters constrained
Δρ_max_ = 0.52 e Å^−3^
Δρ_min_ = −0.47 e Å^−3^
Absolute structure: Flack (1983 ▶), with 3751 Friedel pairs
Flack parameter: 0.46 (5)

### 

Data collection: *CrystalClear* (Rigaku, 2009 ▶); cell refinement: *CrystalClear*; data reduction: *CrystalClear*; program(s) used to solve structure: *SHELXS97* (Sheldrick, 2008 ▶); program(s) used to refine structure: *SHELXL97* (Sheldrick, 2008 ▶); molecular graphics: *SHELXTL* (Sheldrick, 2008 ▶); software used to prepare material for publication: *SHELXTL*.

## Supplementary Material

Crystal structure: contains datablocks global, I. DOI: 10.1107/S1600536810043485/hg2734sup1.cif

Structure factors: contains datablocks I. DOI: 10.1107/S1600536810043485/hg2734Isup2.hkl

Additional supplementary materials:  crystallographic information; 3D view; checkCIF report

## Figures and Tables

**Table 1:** Hydrogen-bond geometry (Å, °) *Cg*1, *Cg*2 and *Cg*3 are the centroids of the C1–C6, C47–C52 and C17–C22 rings, respectively.

*D*—H⋯*A*	*D*—H	H⋯*A*	*D*⋯*A*	*D*—H⋯*A*
C26—H26⋯*Cg*1^i^	0.93	2.86	3.668 (2)	146
C28—H28⋯*Cg*2^ii^	0.93	2.76	3.610 (3)	152
C58—H58⋯*Cg*3^iii^	0.93	2.84	3.588 (3)	138

Symmetry codes: (i) 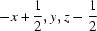; (ii) 
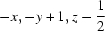
; (iii) 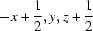.

**Fig. 1.**
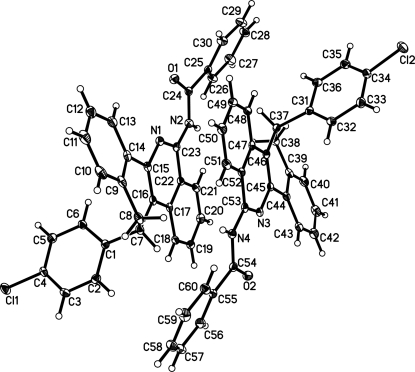
The asymmetric unit of the title compound, (I), with displacement ellipsoids drawn at the 30% probability level.

**Fig. 2.**
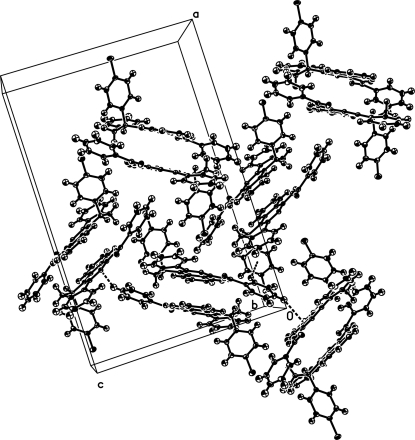
The packing diagram of the title compound. Intermolecular C—H···π interactions are shown as dashed line.

## supplementary crystallographic information

### Comment

Benzo[*c*]phenanthridine derivatives are a class of substances possesing a wide range of pharmacological properties. Many naturally occurring alkaloids that contain a benzo[*c*]phenanthridine ring system and demonstrate interesting biological activities are mentioned in the literature (Clement *et al.*, 2005). For example, antitumor activity (Stermitz *et al.*, 1973, 1975; Fang *et al.*, 1993; Suzuki *et al.*, 1992; Kanzawa *et al.*, 1997; Guo *et al.*, 2007), antimicrobial activity (Nissanka *et al.*, 2001), anti-inflammatory activity (Lenfeld *et al.*, 1981) and antituberculosis activity (Ishikawa, 2001). Because of a strong interest in the biological activities associated with benzo[*c*]phenanthridine compounds, the title compound, (I), was synthesized and its structure is reported here.

The asymmetric unit of (I) contains two independent molecules (Fig. 1). The dihedral angle between the phenyl ring C1–C6 and the benzo[*c*]phenanthridine ring C7–C23/N1 is 87.13 (5)°, and that between the phenyl ring C31–C36 and the benzo[*c*]phenanthridine ring C37–C53/N3 is 79.25 (5)°. The C23—N2—C24—C25 and C53—N4—C54—C55 torision angles are 171.15 (19)° and -171.66 (18)°, respectively. And the intermolecular C—H···π interactions stablize the crystal structure (Fig. 2).

### Experimental

To 15 ml dry dichloromethane, 11-(4-chlorophenyl)-6-amino-11,12-*2H*-benzo[*c*]phenanthridine (0.36 g, 1.0 mmol, prepared according to the reported procedure (Zhang *et al.*, 2008)) and triethylamine (0.18 ml, 1.3 mmol) were added. The mixture was stirred for 30 min at room temperature. A solution of benzoyl chloride (0.17 g, 1.2 mmol) in 3 ml of dry dichloromethane was added dropwise to the above mixture with stirring at room temperature. After completion of the reaction (monitored on TLC), the solvent was removed under vacuum, and the residue was dissolved in 30 ml of water and extracted with dichloromethane. The combined organic phase was dried over sodium sulfate and concentrated under reduced pressure. The resulting crude product was recrystallized from petroleum ether and ethyl acetate. Single crystals of (I) suitable for X-ray diffraction were obtained after slow evaporation of the mother liquor.

### Refinement

All H atoms were placed in calculated positions, with C—H = 0.93, 0.97 or 0.98 Å and N—H = 0.86 Å, and included in the final cycles of refinement using a riding model, with *U*_iso_(H) = 1.2U_eq_(C).

### Crystal data

**Table:**

C_30_H_21_ClN_2_O	*F*(000) = 1920
*M**_r_* = 460.94	*D*_x_ = 1.375 Mg m^−^^3^
Orthorhombic, *P**c**a*2_1_	Mo *K*α radiation, λ = 0.71073 Å
Hall symbol: P 2c -2ac	Cell parameters from 10393 reflections
*a* = 26.567 (5) Å	θ = 1.5–26.1°
*b* = 9.6752 (19) Å	µ = 0.20 mm^−^^1^
*c* = 17.329 (4) Å	*T* = 113 K
*V* = 4454.5 (15) Å^3^	Block, yellow
*Z* = 8	0.20 × 0.18 × 0.10 mm

### Data collection

**Table:**

Rigaku Saturn724 CCD diffractometer	8288 independent reflections
Radiation source: rotating anode	7795 reflections with *I* > 2σ(*I*)
confocal	*R*_int_ = 0.038
ω scans	θ_max_ = 26.1°, θ_min_ = 2.1°
Absorption correction: multi-scan (*CrystalClear*; Rigaku, 2009)	*h* = −28→32
*T*_min_ = 0.961, *T*_max_ = 0.980	*k* = −11→11
27069 measured reflections	*l* = −21→20

### Refinement

**Table:**

Refinement on *F*^2^	Secondary atom site location: difference Fourier map
Least-squares matrix: full	Hydrogen site location: inferred from neighbouring sites
*R*[*F*^2^ > 2σ(*F*^2^)] = 0.040	H-atom parameters constrained
*wR*(*F*^2^) = 0.103	*w* = 1/[σ^2^(*F*_o_^2^) + (0.0607*P*)^2^ + 0.6857*P*] where *P* = (*F*_o_^2^ + 2*F*_c_^2^)/3
*S* = 1.06	(Δ/σ)_max_ = 0.001
8288 reflections	Δρ_max_ = 0.52 e Å^−^^3^
613 parameters	Δρ_min_ = −0.47 e Å^−^^3^
1 restraint	Absolute structure: Flack (1983), with how many Friedel pairs?
Primary atom site location: structure-invariant direct methods	Flack parameter: 0.46 (5)

### Special details

**Table:**

Geometry. All e.s.d.'s (except the e.s.d. in the dihedral angle between two l.s. planes) are estimated using the full covariance matrix. The cell e.s.d.'s are taken into account individually in the estimation of e.s.d.'s in distances, angles and torsion angles; correlations between e.s.d.'s in cell parameters are only used when they are defined by crystal symmetry. An approximate (isotropic) treatment of cell e.s.d.'s is used for estimating e.s.d.'s involving l.s. planes.
Refinement. Refinement of *F*^2^ against ALL reflections. The weighted *R*-factor *wR* and goodness of fit *S* are based on *F*^2^, conventional *R*-factors *R* are based on *F*, with *F* set to zero for negative *F*^2^. The threshold expression of *F*^2^ > σ(*F*^2^) is used only for calculating *R*-factors(gt) *etc*. and is not relevant to the choice of reflections for refinement. *R*-factors based on *F*^2^ are statistically about twice as large as those based on *F*, and *R*-factors based on ALL data will be even larger.

### Fractional atomic coordinates and isotropic or equivalent isotropic displacement parameters (Å^2^)

**Table:**

	*x*	*y*	*z*	*U*_iso_*/*U*_eq_	
Cl1	0.45020 (2)	1.04494 (6)	0.45992 (4)	0.03290 (15)	
Cl2	−0.18314 (2)	0.43588 (7)	0.22904 (4)	0.03730 (16)	
O1	0.15812 (6)	0.44686 (15)	0.20860 (9)	0.0228 (4)	
O2	0.09263 (6)	1.12523 (16)	0.48412 (10)	0.0252 (4)	
N1	0.19235 (6)	0.62652 (18)	0.30450 (11)	0.0184 (4)	
N2	0.14648 (6)	0.68325 (18)	0.19207 (11)	0.0189 (4)	
H2	0.1351	0.7461	0.1616	0.023*	
N3	0.06092 (6)	0.94686 (19)	0.38501 (11)	0.0172 (4)	
N4	0.10706 (6)	0.88891 (19)	0.49698 (11)	0.0184 (4)	
H4	0.1196	0.8257	0.5261	0.022*	
C1	0.29080 (7)	0.8823 (2)	0.47123 (13)	0.0178 (4)	
C2	0.30981 (8)	0.9683 (2)	0.52866 (14)	0.0220 (5)	
H2A	0.2894	0.9922	0.5701	0.026*	
C3	0.35835 (8)	1.0189 (2)	0.52532 (14)	0.0224 (5)	
H3	0.3707	1.0753	0.5644	0.027*	
C4	0.38840 (7)	0.9845 (2)	0.46298 (15)	0.0211 (5)	
C5	0.37101 (9)	0.8999 (2)	0.40441 (14)	0.0242 (5)	
H5	0.3915	0.8774	0.3628	0.029*	
C6	0.32217 (8)	0.8495 (2)	0.40923 (13)	0.0214 (5)	
H6	0.3101	0.7925	0.3702	0.026*	
C7	0.23727 (7)	0.8285 (2)	0.47799 (13)	0.0195 (5)	
H7	0.2170	0.9032	0.5001	0.023*	
C8	0.23284 (8)	0.7046 (2)	0.53287 (14)	0.0237 (5)	
H8A	0.2537	0.7213	0.5778	0.028*	
H8B	0.1983	0.6972	0.5503	0.028*	
C9	0.24816 (8)	0.5706 (2)	0.49689 (14)	0.0234 (5)	
C10	0.27108 (9)	0.4670 (3)	0.53995 (16)	0.0304 (6)	
H10	0.2770	0.4809	0.5923	0.036*	
C11	0.28521 (9)	0.3431 (3)	0.50603 (17)	0.0349 (6)	
H11	0.3012	0.2755	0.5353	0.042*	
C12	0.27543 (9)	0.3201 (3)	0.42832 (17)	0.0314 (6)	
H12	0.2848	0.2370	0.4054	0.038*	
C13	0.25159 (9)	0.4216 (2)	0.38485 (15)	0.0254 (5)	
H13	0.2447	0.4058	0.3330	0.030*	
C14	0.23787 (8)	0.5470 (2)	0.41858 (15)	0.0214 (5)	
C15	0.21420 (8)	0.6601 (2)	0.37485 (13)	0.0188 (5)	
C16	0.21424 (7)	0.7929 (2)	0.40035 (13)	0.0187 (4)	
C17	0.19362 (7)	0.8995 (2)	0.35147 (13)	0.0178 (5)	
C18	0.19268 (8)	1.0404 (2)	0.37309 (14)	0.0202 (5)	
H18	0.2064	1.0672	0.4202	0.024*	
C19	0.17182 (8)	1.1380 (2)	0.32543 (14)	0.0225 (5)	
H19	0.1715	1.2301	0.3409	0.027*	
C20	0.15087 (8)	1.1017 (2)	0.25353 (14)	0.0233 (5)	
H20	0.1373	1.1691	0.2214	0.028*	
C21	0.15077 (8)	0.9652 (2)	0.23163 (14)	0.0208 (5)	
H21	0.1365	0.9399	0.1847	0.025*	
C22	0.17193 (7)	0.8637 (2)	0.27943 (13)	0.0173 (4)	
C23	0.17036 (7)	0.7198 (2)	0.25644 (12)	0.0170 (4)	
C24	0.13982 (8)	0.5481 (2)	0.17403 (13)	0.0195 (5)	
C25	0.10550 (8)	0.5262 (2)	0.10631 (13)	0.0205 (5)	
C26	0.09983 (9)	0.6261 (2)	0.04939 (14)	0.0236 (5)	
H26	0.1183	0.7076	0.0520	0.028*	
C27	0.06682 (10)	0.6050 (3)	−0.01121 (15)	0.0313 (6)	
H27	0.0637	0.6715	−0.0497	0.038*	
C28	0.03835 (9)	0.4848 (3)	−0.01481 (15)	0.0302 (6)	
H28	0.0158	0.4715	−0.0552	0.036*	
C29	0.04355 (10)	0.3858 (3)	0.04136 (16)	0.0316 (6)	
H29	0.0242	0.3058	0.0392	0.038*	
C30	0.07772 (9)	0.4044 (2)	0.10169 (15)	0.0265 (5)	
H30	0.0819	0.3358	0.1387	0.032*	
C31	−0.03808 (7)	0.6800 (2)	0.21873 (13)	0.0186 (4)	
C32	−0.06480 (8)	0.6718 (2)	0.28733 (14)	0.0245 (5)	
H32	−0.0524	0.7160	0.3311	0.029*	
C33	−0.11012 (9)	0.5983 (3)	0.29190 (15)	0.0275 (5)	
H33	−0.1276	0.5915	0.3383	0.033*	
C34	−0.12821 (8)	0.5360 (2)	0.22564 (15)	0.0241 (5)	
C35	−0.10328 (9)	0.5472 (2)	0.15631 (15)	0.0272 (5)	
H35	−0.1167	0.5073	0.1121	0.033*	
C36	−0.05835 (8)	0.6178 (2)	0.15263 (14)	0.0237 (5)	
H36	−0.0413	0.6243	0.1059	0.028*	
C37	0.01430 (7)	0.7465 (2)	0.21222 (13)	0.0191 (5)	
H37	0.0361	0.6759	0.1893	0.023*	
C38	0.01665 (8)	0.8709 (2)	0.15749 (14)	0.0228 (5)	
H38A	0.0508	0.8805	0.1383	0.027*	
H38B	−0.0051	0.8535	0.1136	0.027*	
C39	0.00102 (8)	1.0040 (2)	0.19526 (14)	0.0220 (5)	
C40	−0.02315 (8)	1.1084 (2)	0.15437 (15)	0.0271 (5)	
H40	−0.0309	1.0947	0.1026	0.032*	
C41	−0.03583 (9)	1.2323 (3)	0.18932 (17)	0.0320 (6)	
H41	−0.0521	1.3007	0.1610	0.038*	
C42	−0.02439 (9)	1.2545 (3)	0.26580 (16)	0.0293 (6)	
H42	−0.0331	1.3376	0.2893	0.035*	
C43	0.00040 (8)	1.1519 (2)	0.30821 (15)	0.0226 (5)	
H43	0.0082	1.1668	0.3599	0.027*	
C44	0.01337 (8)	1.0272 (2)	0.27295 (14)	0.0193 (5)	
C45	0.03790 (7)	0.9143 (2)	0.31547 (13)	0.0176 (4)	
C46	0.03744 (7)	0.7808 (2)	0.28962 (12)	0.0169 (4)	
C47	0.05878 (7)	0.6743 (2)	0.33760 (13)	0.0177 (4)	
C48	0.05844 (8)	0.5332 (2)	0.31640 (14)	0.0191 (4)	
H48	0.0441	0.5066	0.2698	0.023*	
C49	0.07913 (8)	0.4348 (2)	0.36405 (14)	0.0223 (5)	
H49	0.0792	0.3428	0.3485	0.027*	
C50	0.10022 (8)	0.4701 (2)	0.43567 (15)	0.0243 (5)	
H50	0.1135	0.4023	0.4678	0.029*	
C51	0.10092 (8)	0.6073 (2)	0.45760 (15)	0.0224 (5)	
H51	0.1151	0.6320	0.5047	0.027*	
C52	0.08047 (7)	0.7101 (2)	0.40953 (13)	0.0167 (4)	
C53	0.08287 (7)	0.8534 (2)	0.43261 (13)	0.0177 (4)	
C54	0.11219 (8)	1.0245 (2)	0.51734 (14)	0.0199 (5)	
C55	0.14664 (8)	1.0465 (2)	0.58493 (13)	0.0205 (5)	
C56	0.17109 (8)	1.1721 (2)	0.59367 (15)	0.0270 (5)	
H56	0.1656	1.2427	0.5583	0.032*	
C57	0.20390 (9)	1.1930 (3)	0.65529 (17)	0.0330 (6)	
H57	0.2203	1.2774	0.6609	0.040*	
C58	0.21214 (9)	1.0887 (3)	0.70806 (15)	0.0329 (6)	
H58	0.2339	1.1030	0.7493	0.039*	
C59	0.18785 (9)	0.9620 (3)	0.69954 (16)	0.0315 (6)	
H59	0.1934	0.8914	0.7350	0.038*	
C60	0.15552 (8)	0.9418 (2)	0.63837 (14)	0.0248 (5)	
H60	0.1394	0.8571	0.6327	0.030*	

### Atomic displacement parameters (Å^2^)

**Table:**

	*U*^11^	*U*^22^	*U*^33^	*U*^12^	*U*^13^	*U*^23^
Cl1	0.0214 (3)	0.0420 (3)	0.0352 (3)	−0.0113 (2)	0.0024 (2)	−0.0036 (3)
Cl2	0.0298 (3)	0.0409 (3)	0.0412 (4)	−0.0124 (3)	−0.0001 (3)	−0.0072 (3)
O1	0.0246 (8)	0.0233 (8)	0.0205 (9)	0.0035 (6)	−0.0023 (6)	0.0008 (7)
O2	0.0262 (8)	0.0221 (8)	0.0273 (10)	0.0040 (7)	−0.0061 (7)	−0.0017 (7)
N1	0.0178 (9)	0.0214 (9)	0.0159 (10)	0.0002 (7)	−0.0010 (7)	0.0020 (8)
N2	0.0204 (9)	0.0182 (9)	0.0181 (10)	−0.0006 (7)	−0.0014 (7)	0.0033 (8)
N3	0.0133 (8)	0.0200 (9)	0.0182 (10)	0.0004 (7)	−0.0010 (7)	0.0020 (7)
N4	0.0175 (9)	0.0212 (9)	0.0164 (10)	0.0017 (7)	−0.0013 (7)	0.0014 (8)
C1	0.0170 (10)	0.0178 (10)	0.0185 (12)	0.0009 (8)	−0.0018 (8)	0.0021 (9)
C2	0.0232 (11)	0.0242 (12)	0.0188 (12)	0.0023 (9)	0.0016 (9)	0.0000 (9)
C3	0.0252 (11)	0.0206 (11)	0.0216 (12)	−0.0029 (9)	−0.0062 (9)	−0.0013 (9)
C4	0.0146 (10)	0.0225 (11)	0.0262 (12)	−0.0044 (8)	−0.0030 (9)	0.0027 (10)
C5	0.0230 (11)	0.0294 (12)	0.0203 (12)	0.0001 (10)	0.0043 (9)	−0.0017 (10)
C6	0.0187 (11)	0.0277 (12)	0.0179 (12)	−0.0023 (9)	−0.0014 (8)	−0.0034 (10)
C7	0.0166 (10)	0.0252 (11)	0.0166 (12)	−0.0004 (8)	−0.0025 (8)	0.0003 (9)
C8	0.0192 (11)	0.0334 (12)	0.0185 (12)	−0.0058 (9)	−0.0029 (8)	0.0053 (10)
C9	0.0168 (10)	0.0267 (11)	0.0268 (14)	−0.0075 (9)	−0.0048 (9)	0.0079 (10)
C10	0.0277 (12)	0.0330 (14)	0.0304 (15)	−0.0097 (11)	−0.0121 (10)	0.0123 (11)
C11	0.0298 (13)	0.0287 (13)	0.0462 (18)	−0.0045 (11)	−0.0154 (11)	0.0164 (12)
C12	0.0237 (12)	0.0239 (12)	0.0464 (17)	−0.0008 (10)	−0.0070 (11)	0.0064 (12)
C13	0.0219 (11)	0.0246 (11)	0.0296 (14)	−0.0028 (10)	−0.0056 (9)	0.0034 (10)
C14	0.0134 (10)	0.0238 (12)	0.0271 (13)	−0.0019 (8)	−0.0027 (8)	0.0079 (10)
C15	0.0141 (10)	0.0231 (11)	0.0190 (12)	−0.0017 (8)	−0.0006 (8)	0.0035 (9)
C16	0.0121 (9)	0.0276 (12)	0.0164 (11)	−0.0010 (8)	0.0003 (8)	0.0015 (9)
C17	0.0111 (10)	0.0211 (11)	0.0213 (12)	−0.0013 (8)	0.0011 (8)	0.0023 (9)
C18	0.0147 (10)	0.0247 (11)	0.0211 (12)	−0.0026 (9)	0.0000 (8)	−0.0033 (9)
C19	0.0192 (10)	0.0205 (11)	0.0278 (13)	−0.0011 (9)	0.0020 (9)	−0.0015 (10)
C20	0.0215 (11)	0.0232 (11)	0.0250 (13)	0.0023 (9)	−0.0015 (9)	0.0072 (10)
C21	0.0183 (10)	0.0249 (11)	0.0193 (12)	−0.0032 (8)	−0.0029 (9)	0.0016 (10)
C22	0.0139 (9)	0.0225 (11)	0.0154 (11)	−0.0016 (8)	0.0004 (8)	−0.0003 (9)
C23	0.0147 (10)	0.0205 (11)	0.0158 (11)	0.0009 (8)	0.0025 (8)	0.0037 (9)
C24	0.0159 (10)	0.0234 (11)	0.0191 (12)	−0.0016 (9)	0.0039 (8)	−0.0012 (9)
C25	0.0174 (10)	0.0266 (12)	0.0175 (12)	0.0021 (9)	0.0012 (8)	−0.0052 (9)
C26	0.0275 (12)	0.0222 (11)	0.0212 (13)	−0.0062 (9)	−0.0015 (9)	0.0011 (10)
C27	0.0363 (14)	0.0365 (14)	0.0212 (13)	−0.0051 (11)	−0.0066 (10)	0.0045 (11)
C28	0.0283 (12)	0.0366 (14)	0.0256 (14)	−0.0045 (11)	−0.0071 (9)	−0.0063 (11)
C29	0.0342 (13)	0.0285 (13)	0.0323 (15)	−0.0092 (11)	−0.0042 (10)	−0.0057 (11)
C30	0.0315 (13)	0.0239 (12)	0.0241 (13)	−0.0022 (10)	−0.0023 (10)	−0.0001 (10)
C31	0.0187 (10)	0.0164 (10)	0.0207 (12)	0.0038 (8)	−0.0025 (8)	0.0007 (9)
C32	0.0240 (11)	0.0294 (12)	0.0202 (13)	−0.0020 (10)	−0.0008 (9)	−0.0061 (10)
C33	0.0229 (12)	0.0335 (13)	0.0261 (14)	−0.0010 (10)	0.0030 (9)	−0.0019 (11)
C34	0.0183 (10)	0.0248 (12)	0.0292 (13)	−0.0010 (9)	−0.0028 (10)	−0.0018 (11)
C35	0.0260 (12)	0.0283 (12)	0.0275 (14)	0.0016 (10)	−0.0072 (10)	−0.0068 (10)
C36	0.0284 (12)	0.0250 (12)	0.0177 (12)	−0.0004 (9)	0.0001 (9)	−0.0028 (10)
C37	0.0170 (10)	0.0228 (11)	0.0174 (12)	0.0028 (8)	−0.0001 (8)	−0.0019 (9)
C38	0.0225 (11)	0.0275 (12)	0.0182 (12)	−0.0035 (9)	−0.0031 (9)	0.0030 (10)
C39	0.0175 (10)	0.0253 (11)	0.0233 (12)	−0.0061 (9)	−0.0045 (9)	0.0037 (10)
C40	0.0247 (12)	0.0314 (13)	0.0251 (13)	−0.0031 (10)	−0.0074 (9)	0.0066 (11)
C41	0.0250 (12)	0.0249 (12)	0.0462 (17)	−0.0007 (10)	−0.0130 (11)	0.0132 (12)
C42	0.0242 (12)	0.0208 (12)	0.0430 (16)	0.0013 (10)	−0.0045 (11)	0.0054 (11)
C43	0.0172 (10)	0.0223 (11)	0.0283 (13)	−0.0021 (9)	−0.0029 (9)	0.0023 (10)
C44	0.0151 (10)	0.0199 (11)	0.0228 (13)	−0.0006 (8)	−0.0028 (8)	0.0041 (9)
C45	0.0116 (9)	0.0220 (10)	0.0193 (12)	−0.0002 (8)	0.0001 (8)	0.0002 (9)
C46	0.0141 (10)	0.0217 (11)	0.0150 (11)	0.0002 (8)	0.0011 (8)	0.0022 (9)
C47	0.0112 (10)	0.0225 (11)	0.0193 (12)	−0.0008 (8)	0.0042 (8)	0.0028 (9)
C48	0.0180 (10)	0.0207 (11)	0.0185 (12)	−0.0024 (8)	0.0009 (8)	−0.0024 (9)
C49	0.0207 (11)	0.0182 (11)	0.0280 (14)	−0.0015 (9)	0.0012 (9)	−0.0013 (10)
C50	0.0212 (11)	0.0232 (12)	0.0285 (14)	0.0015 (9)	−0.0033 (9)	0.0073 (10)
C51	0.0203 (10)	0.0266 (11)	0.0202 (12)	−0.0029 (9)	−0.0022 (9)	0.0028 (10)
C52	0.0134 (9)	0.0182 (10)	0.0186 (11)	−0.0020 (8)	0.0017 (8)	0.0011 (9)
C53	0.0120 (9)	0.0250 (11)	0.0160 (11)	0.0002 (8)	0.0018 (8)	0.0035 (9)
C54	0.0172 (10)	0.0235 (12)	0.0190 (12)	0.0013 (9)	0.0017 (8)	−0.0004 (9)
C55	0.0157 (10)	0.0249 (12)	0.0208 (13)	0.0010 (9)	0.0006 (8)	−0.0048 (9)
C56	0.0253 (12)	0.0260 (12)	0.0297 (14)	0.0034 (9)	−0.0049 (10)	−0.0023 (11)
C57	0.0324 (13)	0.0307 (13)	0.0361 (16)	−0.0045 (11)	−0.0086 (11)	−0.0088 (12)
C58	0.0282 (13)	0.0454 (15)	0.0251 (14)	0.0004 (11)	−0.0089 (10)	−0.0062 (12)
C59	0.0319 (13)	0.0418 (15)	0.0209 (13)	0.0014 (11)	−0.0030 (10)	0.0065 (11)
C60	0.0237 (11)	0.0298 (12)	0.0208 (13)	−0.0041 (9)	−0.0008 (9)	0.0001 (11)

### Geometric parameters (Å, °)

**Table:**

Cl1—C4	1.744 (2)	C27—C28	1.388 (4)
Cl2—C34	1.752 (2)	C27—H27	0.9300
O1—C24	1.247 (3)	C28—C29	1.373 (4)
O2—C54	1.245 (3)	C28—H28	0.9300
N1—C23	1.360 (3)	C29—C30	1.396 (4)
N1—C15	1.389 (3)	C29—H29	0.9300
N2—C23	1.331 (3)	C30—H30	0.9300
N2—C24	1.356 (3)	C31—C32	1.387 (3)
N2—H2	0.8600	C31—C36	1.401 (3)
N3—C53	1.356 (3)	C31—C37	1.537 (3)
N3—C45	1.388 (3)	C32—C33	1.401 (3)
N4—C53	1.332 (3)	C32—H32	0.9300
N4—C54	1.366 (3)	C33—C34	1.383 (4)
N4—H4	0.8600	C33—H33	0.9300
C1—C2	1.392 (3)	C34—C35	1.376 (4)
C1—C6	1.396 (3)	C35—C36	1.377 (3)
C1—C7	1.519 (3)	C35—H35	0.9300
C2—C3	1.380 (3)	C36—H36	0.9300
C2—H2A	0.9300	C37—C46	1.512 (3)
C3—C4	1.384 (3)	C37—C38	1.533 (3)
C3—H3	0.9300	C37—H37	0.9800
C4—C5	1.383 (3)	C38—C39	1.503 (3)
C5—C6	1.389 (3)	C38—H38A	0.9700
C5—H5	0.9300	C38—H38B	0.9700
C6—H6	0.9300	C39—C40	1.390 (3)
C7—C16	1.517 (3)	C39—C44	1.404 (3)
C7—C8	1.534 (3)	C40—C41	1.385 (4)
C7—H7	0.9800	C40—H40	0.9300
C8—C9	1.495 (3)	C41—C42	1.377 (4)
C8—H8A	0.9700	C41—H41	0.9300
C8—H8B	0.9700	C42—C43	1.400 (3)
C9—C10	1.390 (3)	C42—H42	0.9300
C9—C14	1.403 (3)	C43—C44	1.395 (3)
C10—C11	1.387 (4)	C43—H43	0.9300
C10—H10	0.9300	C44—C45	1.470 (3)
C11—C12	1.389 (4)	C45—C46	1.367 (3)
C11—H11	0.9300	C46—C47	1.440 (3)
C12—C13	1.391 (3)	C47—C48	1.414 (3)
C12—H12	0.9300	C47—C52	1.416 (3)
C13—C14	1.395 (3)	C48—C49	1.375 (3)
C13—H13	0.9300	C48—H48	0.9300
C14—C15	1.472 (3)	C49—C50	1.404 (3)
C15—C16	1.359 (3)	C49—H49	0.9300
C16—C17	1.443 (3)	C50—C51	1.381 (3)
C17—C18	1.414 (3)	C50—H50	0.9300
C17—C22	1.418 (3)	C51—C52	1.407 (3)
C18—C19	1.372 (3)	C51—H51	0.9300
C18—H18	0.9300	C52—C53	1.444 (3)
C19—C20	1.409 (3)	C54—C55	1.502 (3)
C19—H19	0.9300	C55—C56	1.386 (3)
C20—C21	1.374 (3)	C55—C60	1.393 (3)
C20—H20	0.9300	C56—C57	1.393 (3)
C21—C22	1.402 (3)	C56—H56	0.9300
C21—H21	0.9300	C57—C58	1.379 (4)
C22—C23	1.449 (3)	C57—H57	0.9300
C24—C25	1.501 (3)	C58—C59	1.393 (4)
C25—C26	1.389 (3)	C58—H58	0.9300
C25—C30	1.392 (3)	C59—C60	1.379 (3)
C26—C27	1.383 (3)	C59—H59	0.9300
C26—H26	0.9300	C60—H60	0.9300
			
C23—N1—C15	124.20 (18)	C30—C29—H29	119.8
C23—N2—C24	120.75 (19)	C25—C30—C29	119.8 (2)
C23—N2—H2	119.6	C25—C30—H30	120.1
C24—N2—H2	119.6	C29—C30—H30	120.1
C53—N3—C45	124.49 (19)	C32—C31—C36	118.6 (2)
C53—N4—C54	120.82 (19)	C32—C31—C37	123.4 (2)
C53—N4—H4	119.6	C36—C31—C37	117.9 (2)
C54—N4—H4	119.6	C31—C32—C33	121.2 (2)
C2—C1—C6	117.99 (19)	C31—C32—H32	119.4
C2—C1—C7	119.32 (19)	C33—C32—H32	119.4
C6—C1—C7	122.69 (19)	C34—C33—C32	118.2 (2)
C3—C2—C1	121.4 (2)	C34—C33—H33	120.9
C3—C2—H2A	119.3	C32—C33—H33	120.9
C1—C2—H2A	119.3	C35—C34—C33	121.5 (2)
C2—C3—C4	119.1 (2)	C35—C34—Cl2	118.28 (18)
C2—C3—H3	120.4	C33—C34—Cl2	120.16 (19)
C4—C3—H3	120.4	C34—C35—C36	119.8 (2)
C5—C4—C3	121.49 (19)	C34—C35—H35	120.1
C5—C4—Cl1	119.38 (18)	C36—C35—H35	120.1
C3—C4—Cl1	119.10 (18)	C35—C36—C31	120.6 (2)
C4—C5—C6	118.4 (2)	C35—C36—H36	119.7
C4—C5—H5	120.8	C31—C36—H36	119.7
C6—C5—H5	120.8	C46—C37—C38	111.10 (18)
C5—C6—C1	121.6 (2)	C46—C37—C31	113.25 (18)
C5—C6—H6	119.2	C38—C37—C31	114.26 (17)
C1—C6—H6	119.2	C46—C37—H37	105.8
C16—C7—C1	112.76 (18)	C38—C37—H37	105.8
C16—C7—C8	109.98 (18)	C31—C37—H37	105.8
C1—C7—C8	112.78 (17)	C39—C38—C37	113.1 (2)
C16—C7—H7	107.0	C39—C38—H38A	109.0
C1—C7—H7	107.0	C37—C38—H38A	109.0
C8—C7—H7	107.0	C39—C38—H38B	109.0
C9—C8—C7	113.4 (2)	C37—C38—H38B	109.0
C9—C8—H8A	108.9	H38A—C38—H38B	107.8
C7—C8—H8A	108.9	C40—C39—C44	118.7 (2)
C9—C8—H8B	108.9	C40—C39—C38	121.9 (2)
C7—C8—H8B	108.9	C44—C39—C38	119.3 (2)
H8A—C8—H8B	107.7	C41—C40—C39	121.2 (2)
C10—C9—C14	119.2 (2)	C41—C40—H40	119.4
C10—C9—C8	121.4 (2)	C39—C40—H40	119.4
C14—C9—C8	119.4 (2)	C42—C41—C40	120.1 (2)
C11—C10—C9	121.0 (3)	C42—C41—H41	119.9
C11—C10—H10	119.5	C40—C41—H41	119.9
C9—C10—H10	119.5	C41—C42—C43	119.9 (2)
C10—C11—C12	119.9 (2)	C41—C42—H42	120.0
C10—C11—H11	120.0	C43—C42—H42	120.0
C12—C11—H11	120.0	C44—C43—C42	120.0 (2)
C11—C12—C13	119.8 (2)	C44—C43—H43	120.0
C11—C12—H12	120.1	C42—C43—H43	120.0
C13—C12—H12	120.1	C43—C44—C39	120.0 (2)
C12—C13—C14	120.4 (2)	C43—C44—C45	122.2 (2)
C12—C13—H13	119.8	C39—C44—C45	117.7 (2)
C14—C13—H13	119.8	C46—C45—N3	120.2 (2)
C13—C14—C9	119.7 (2)	C46—C45—C44	122.3 (2)
C13—C14—C15	122.8 (2)	N3—C45—C44	117.49 (19)
C9—C14—C15	117.4 (2)	C45—C46—C47	118.9 (2)
C16—C15—N1	120.47 (19)	C45—C46—C37	120.10 (19)
C16—C15—C14	122.3 (2)	C47—C46—C37	121.01 (19)
N1—C15—C14	117.17 (19)	C48—C47—C52	117.9 (2)
C15—C16—C17	119.0 (2)	C48—C47—C46	122.6 (2)
C15—C16—C7	120.2 (2)	C52—C47—C46	119.6 (2)
C17—C16—C7	120.8 (2)	C49—C48—C47	120.7 (2)
C18—C17—C22	117.5 (2)	C49—C48—H48	119.7
C18—C17—C16	122.7 (2)	C47—C48—H48	119.7
C22—C17—C16	119.75 (19)	C48—C49—C50	121.5 (2)
C19—C18—C17	120.8 (2)	C48—C49—H49	119.3
C19—C18—H18	119.6	C50—C49—H49	119.3
C17—C18—H18	119.6	C51—C50—C49	118.9 (2)
C18—C19—C20	121.3 (2)	C51—C50—H50	120.6
C18—C19—H19	119.3	C49—C50—H50	120.6
C20—C19—H19	119.3	C50—C51—C52	120.8 (2)
C21—C20—C19	119.0 (2)	C50—C51—H51	119.6
C21—C20—H20	120.5	C52—C51—H51	119.6
C19—C20—H20	120.5	C51—C52—C47	120.4 (2)
C20—C21—C22	120.6 (2)	C51—C52—C53	119.8 (2)
C20—C21—H21	119.7	C47—C52—C53	119.77 (19)
C22—C21—H21	119.7	N4—C53—N3	123.0 (2)
C21—C22—C17	120.8 (2)	N4—C53—C52	120.06 (19)
C21—C22—C23	119.9 (2)	N3—C53—C52	116.92 (19)
C17—C22—C23	119.27 (19)	O2—C54—N4	126.2 (2)
N2—C23—N1	122.8 (2)	O2—C54—C55	120.3 (2)
N2—C23—C22	119.95 (19)	N4—C54—C55	113.47 (19)
N1—C23—C22	117.17 (19)	C56—C55—C60	119.1 (2)
O1—C24—N2	126.6 (2)	C56—C55—C54	119.7 (2)
O1—C24—C25	120.09 (19)	C60—C55—C54	121.3 (2)
N2—C24—C25	113.32 (19)	C55—C56—C57	120.3 (2)
C26—C25—C30	119.4 (2)	C55—C56—H56	119.9
C26—C25—C24	121.5 (2)	C57—C56—H56	119.9
C30—C25—C24	119.1 (2)	C58—C57—C56	120.1 (2)
C27—C26—C25	120.3 (2)	C58—C57—H57	120.0
C27—C26—H26	119.8	C56—C57—H57	120.0
C25—C26—H26	119.8	C57—C58—C59	120.0 (2)
C26—C27—C28	120.2 (2)	C57—C58—H58	120.0
C26—C27—H27	119.9	C59—C58—H58	120.0
C28—C27—H27	119.9	C60—C59—C58	119.7 (2)
C29—C28—C27	119.8 (2)	C60—C59—H59	120.2
C29—C28—H28	120.1	C58—C59—H59	120.2
C27—C28—H28	120.1	C59—C60—C55	120.9 (2)
C28—C29—C30	120.4 (2)	C59—C60—H60	119.5
C28—C29—H29	119.8	C55—C60—H60	119.5
			
C6—C1—C2—C3	−0.7 (3)	C36—C31—C32—C33	2.7 (3)
C7—C1—C2—C3	179.5 (2)	C37—C31—C32—C33	−173.6 (2)
C1—C2—C3—C4	0.8 (3)	C31—C32—C33—C34	−1.3 (4)
C2—C3—C4—C5	−0.5 (3)	C32—C33—C34—C35	−1.2 (4)
C2—C3—C4—Cl1	−178.46 (18)	C32—C33—C34—Cl2	176.98 (18)
C3—C4—C5—C6	0.1 (4)	C33—C34—C35—C36	2.3 (4)
Cl1—C4—C5—C6	178.02 (18)	Cl2—C34—C35—C36	−175.94 (18)
C4—C5—C6—C1	0.1 (3)	C34—C35—C36—C31	−0.8 (3)
C2—C1—C6—C5	0.3 (3)	C32—C31—C36—C35	−1.6 (3)
C7—C1—C6—C5	−179.9 (2)	C37—C31—C36—C35	174.9 (2)
C2—C1—C7—C16	154.6 (2)	C32—C31—C37—C46	9.5 (3)
C6—C1—C7—C16	−25.2 (3)	C36—C31—C37—C46	−166.81 (19)
C2—C1—C7—C8	−80.1 (3)	C32—C31—C37—C38	−119.0 (2)
C6—C1—C7—C8	100.1 (2)	C36—C31—C37—C38	64.6 (3)
C16—C7—C8—C9	46.6 (2)	C46—C37—C38—C39	−45.4 (2)
C1—C7—C8—C9	−80.2 (2)	C31—C37—C38—C39	84.2 (2)
C7—C8—C9—C10	146.8 (2)	C37—C38—C39—C40	−147.5 (2)
C7—C8—C9—C14	−35.2 (3)	C37—C38—C39—C44	35.8 (3)
C14—C9—C10—C11	2.0 (3)	C44—C39—C40—C41	−1.2 (3)
C8—C9—C10—C11	−179.9 (2)	C38—C39—C40—C41	−177.8 (2)
C9—C10—C11—C12	−1.5 (4)	C39—C40—C41—C42	0.3 (4)
C10—C11—C12—C13	0.1 (4)	C40—C41—C42—C43	0.4 (4)
C11—C12—C13—C14	0.6 (4)	C41—C42—C43—C44	−0.1 (3)
C12—C13—C14—C9	−0.1 (3)	C42—C43—C44—C39	−0.8 (3)
C12—C13—C14—C15	177.8 (2)	C42—C43—C44—C45	−177.9 (2)
C10—C9—C14—C13	−1.2 (3)	C40—C39—C44—C43	1.4 (3)
C8—C9—C14—C13	−179.3 (2)	C38—C39—C44—C43	178.2 (2)
C10—C9—C14—C15	−179.2 (2)	C40—C39—C44—C45	178.7 (2)
C8—C9—C14—C15	2.7 (3)	C38—C39—C44—C45	−4.6 (3)
C23—N1—C15—C16	0.6 (3)	C53—N3—C45—C46	−1.8 (3)
C23—N1—C15—C14	−178.02 (19)	C53—N3—C45—C44	176.6 (2)
C13—C14—C15—C16	−160.3 (2)	C43—C44—C45—C46	160.3 (2)
C9—C14—C15—C16	17.6 (3)	C39—C44—C45—C46	−16.9 (3)
C13—C14—C15—N1	18.3 (3)	C43—C44—C45—N3	−18.1 (3)
C9—C14—C15—N1	−163.79 (19)	C39—C44—C45—N3	164.72 (19)
N1—C15—C16—C17	−3.2 (3)	N3—C45—C46—C47	3.3 (3)
C14—C15—C16—C17	175.35 (19)	C44—C45—C46—C47	−175.03 (19)
N1—C15—C16—C7	178.80 (18)	N3—C45—C46—C37	−177.39 (18)
C14—C15—C16—C7	−2.6 (3)	C44—C45—C46—C37	4.3 (3)
C1—C7—C16—C15	97.6 (2)	C38—C37—C46—C45	27.0 (3)
C8—C7—C16—C15	−29.2 (3)	C31—C37—C46—C45	−103.2 (2)
C1—C7—C16—C17	−80.4 (2)	C38—C37—C46—C47	−153.72 (18)
C8—C7—C16—C17	152.82 (19)	C31—C37—C46—C47	76.1 (2)
C15—C16—C17—C18	−179.3 (2)	C45—C46—C47—C48	177.9 (2)
C7—C16—C17—C18	−1.3 (3)	C37—C46—C47—C48	−1.4 (3)
C15—C16—C17—C22	2.5 (3)	C45—C46—C47—C52	−1.1 (3)
C7—C16—C17—C22	−179.49 (18)	C37—C46—C47—C52	179.57 (18)
C22—C17—C18—C19	−0.5 (3)	C52—C47—C48—C49	−0.8 (3)
C16—C17—C18—C19	−178.7 (2)	C46—C47—C48—C49	−179.8 (2)
C17—C18—C19—C20	−0.2 (3)	C47—C48—C49—C50	1.4 (3)
C18—C19—C20—C21	1.0 (3)	C48—C49—C50—C51	−1.3 (3)
C19—C20—C21—C22	−1.1 (3)	C49—C50—C51—C52	0.6 (3)
C20—C21—C22—C17	0.3 (3)	C50—C51—C52—C47	0.0 (3)
C20—C21—C22—C23	178.4 (2)	C50—C51—C52—C53	−178.3 (2)
C18—C17—C22—C21	0.4 (3)	C48—C47—C52—C51	0.1 (3)
C16—C17—C22—C21	178.74 (19)	C46—C47—C52—C51	179.20 (19)
C18—C17—C22—C23	−177.63 (19)	C48—C47—C52—C53	178.34 (18)
C16—C17—C22—C23	0.7 (3)	C46—C47—C52—C53	−2.6 (3)
C24—N2—C23—N1	4.8 (3)	C54—N4—C53—N3	−1.9 (3)
C24—N2—C23—C22	−172.68 (19)	C54—N4—C53—C52	176.19 (19)
C15—N1—C23—N2	−174.92 (19)	C45—N3—C53—N4	176.24 (19)
C15—N1—C23—C22	2.6 (3)	C45—N3—C53—C52	−1.9 (3)
C21—C22—C23—N2	−3.6 (3)	C51—C52—C53—N4	4.0 (3)
C17—C22—C23—N2	174.47 (19)	C47—C52—C53—N4	−174.20 (19)
C21—C22—C23—N1	178.78 (19)	C51—C52—C53—N3	−177.75 (19)
C17—C22—C23—N1	−3.1 (3)	C47—C52—C53—N3	4.0 (3)
C23—N2—C24—O1	−7.2 (3)	C53—N4—C54—O2	6.1 (3)
C23—N2—C24—C25	171.15 (19)	C53—N4—C54—C55	−171.66 (18)
O1—C24—C25—C26	−154.9 (2)	O2—C54—C55—C56	−23.3 (3)
N2—C24—C25—C26	26.6 (3)	N4—C54—C55—C56	154.6 (2)
O1—C24—C25—C30	26.9 (3)	O2—C54—C55—C60	158.2 (2)
N2—C24—C25—C30	−151.6 (2)	N4—C54—C55—C60	−23.9 (3)
C30—C25—C26—C27	0.1 (4)	C60—C55—C56—C57	−0.2 (3)
C24—C25—C26—C27	−178.1 (2)	C54—C55—C56—C57	−178.8 (2)
C25—C26—C27—C28	1.3 (4)	C55—C56—C57—C58	−0.1 (4)
C26—C27—C28—C29	−1.0 (4)	C56—C57—C58—C59	0.3 (4)
C27—C28—C29—C30	−0.7 (4)	C57—C58—C59—C60	−0.2 (4)
C26—C25—C30—C29	−1.8 (4)	C58—C59—C60—C55	−0.2 (4)
C24—C25—C30—C29	176.4 (2)	C56—C55—C60—C59	0.4 (4)
C28—C29—C30—C25	2.1 (4)	C54—C55—C60—C59	178.9 (2)

### Hydrogen-bond geometry (Å, °)

**Table:**

*D*—H···*A*	*D*—H	H···*A*	*D*···*A*	*D*—H···*A*
C26—H26···Cg1^i^	0.93	2.86	3.668 (2)	146
C28—H28···Cg2^ii^	0.93	2.76	3.610 (3)	152
C58—H58···Cg3^iii^	0.93	2.84	3.588 (3)	138

Symmetry codes: (i) −*x*+1/2, *y*, *z*−1/2; (ii) −*x*, −*y*+1, *z*−1/2; (iii) −*x*+1/2, *y*, *z*+1/2.
